# High-Normal Serum Magnesium and Hypermagnesemia Are Associated With Increased 30-Day In-Hospital Mortality: A Retrospective Cohort Study

**DOI:** 10.3389/fcvm.2021.625133

**Published:** 2021-02-10

**Authors:** Liao Tan, Qian Xu, Chan Li, Jie Liu, Ruizheng Shi

**Affiliations:** ^1^Department of Cardiology, The Third Xiangya Hospital, Central South University, Changsha, China; ^2^Department of Cardiovascular Medicine, Xiangya Hospital, Central South University, Changsha, China; ^3^Department of Cardiovascular Surgery, Xiangya Hospital, Central South University, Changsha, China

**Keywords:** serum magnesium, ICU, acute myocardial infarction, in-hospital mortality, prediction

## Abstract

**Background:** Magnesium, the fourth most abundant mineral nutrient in our body, plays a critical role in regulating ion channels and energy generation, intracardiac conduction, and myocardial contraction. In this study, we assessed the association of admission serum magnesium level with all-cause in-hospital mortality in critically ill patients with acute myocardial infarction (AMI).

**Methods:** Clinical data were extracted from the eICU Collaborative Research Database (eICU-CRD). Only the data for the first intensive care unit (ICU) admission of each patient were used, and baseline data were extracted within 24 h after ICU admission. Logistic regression, Cox regression, and subgroup analyses were conducted to determine the relationship between admission serum magnesium level and 30-day in-hospital mortality in ICU patients with AMI.

**Results:** A total of 9,005 eligible patients were included. In the logistic regression analysis, serum magnesium at 2.2 to ≤2.4 and >2.4 mg/dl levels were both significant predictors of all-cause in-hospital mortality in AMI patients. Moreover, serum magnesium of 2.2 to ≤2.4 mg/dl showed higher risk of in-hospital mortality than magnesium of >2.4 mg/dl (adjusted odds ratio, 1.63 vs. 1.39). The Cox regression analysis yielded similar results (adjusted hazard ratio, 1.36 vs. 1.25).

**Conclusions:** High-normal serum magnesium and hypermagnesemia may be useful and easier predictors for 30-day in-hospital mortality in critically ill patients with AMI.

## Introduction

With the development of therapeutic strategies, the survival rate and survival time of patients with acute myocardial infarction (AMI) increased year by year. However, the in-hospital mortality rate of AMI is still around 8% (1–4), which is largely due to the complicated and rapid progression, high incidence of complications of AMI, and delayed medical intervention (5). Hence, in order to predict in-hospital mortality in early stage, more early-stage and simple biomarkers should be explored to predict the risk of in-hospital mortality in patients with AMI.

The result from coronary angiography and electrocardiogram (ECG) are verified as the most popular prognostic determinant in AMI. However, an increasing number of studies show that mortality of AMI patients is associated not only with the radiological appearance but also with biochemical results, such as high-sensitivity troponin T (Hs-TnT) and N-terminal pro B-type natriuretic peptide (NT-proBNP) (6). However, the detection of many specific biomarkers is time-consuming and not cost-effective. Hence, more cheap and routine biochemical variables for prognostic prediction should be explored.

Serum magnesium levels are critical for cardio-physiological regulatory mechanisms including regulating ion channels and energy generation, intracardiac conduction, and myocardial contraction (7). Magnesium plays an important role in regulating vascular tone, atherogenesis and thrombosis, proliferation and migration of vascular smooth muscle cells and endothelial cells, and vascular calcification, etc. (7). In addition, serum magnesium measurement is typically enrolled in routine biochemical tests of electrolyte levels (8). Moreover, the detection and analysis of serum magnesium are simple and low cost. Therefore, serum magnesium is measured routinely for most patients admitted in the hospital. Previous studies proved that serum magnesium levels decreased in patients with AMI and that low magnesium levels are associated with malignant arrhythmias (9, 10). However, whether serum magnesium levels at intensive care unit (ICU) admission are related to in-hospital mortality still needs to be further validated.

Electronic health record (EHR) system is defined as a kind of medical information storage system that uses various technologies in order to construct, manage, store, and share a mass of EHR (11). Up to now, EHR data have been recognized as a promising resource for finding risk factors of diseases (11). eICU Collaborative Research Database (eICU-CRD) is a large-scale multicenter open-access ICU EHR (12). The EHR of patients who were admitted to one of 335 units at 208 hospitals in the USA was included in this database, including admission diagnosis, demographics, patient history, vital signs, laboratory examinations, medications, and care plan information. eICU-CRD was widely used to explore risk factors of sepsis and acute kidney injury (AKI) (13, 14). However, few studies focused on finding predictors of mortality of AMI. Our study is the first to use eICU-CRD to explore the relationship between admission serum magnesium and in-hospital mortality of AMI patients.

In this study, clinical data of AMI patients were extracted from the eICU-CRD, and admission serum magnesium levels were divided into five groups (<1.8 mg/dl; ≥1.8, <2.0 mg/dl; ≥2.0, ≤2.2 mg/dl; >2.2, ≤2.4 mg/dl; and >2.4 mg/dl). The reference for serum magnesium levels of ≥2.0, ≤2.2 mg/dl was selected on the basis of reviewing relevant literature (15). Then logistic regression and Cox regression analysis, as well as subgroup analyses, were conducted subsequently. Through these analyses, the association between the admission serum magnesium with the in-hospital mortality of AMI patients could be confirmed.

## Methods

### Database Source

The eICU-CRD is a large, multicenter, open-access critical care database. It includes more than 200,000 patients with ICU admissions. To obtain access to this database, author Tan passed the Protecting Human Research Participants exam and obtained certification (certification number: 35950815). This project was approved by the institutional review boards of the Massachusetts Institute of Technology (MIT).

### Study Population Selection

**Figure 1:**
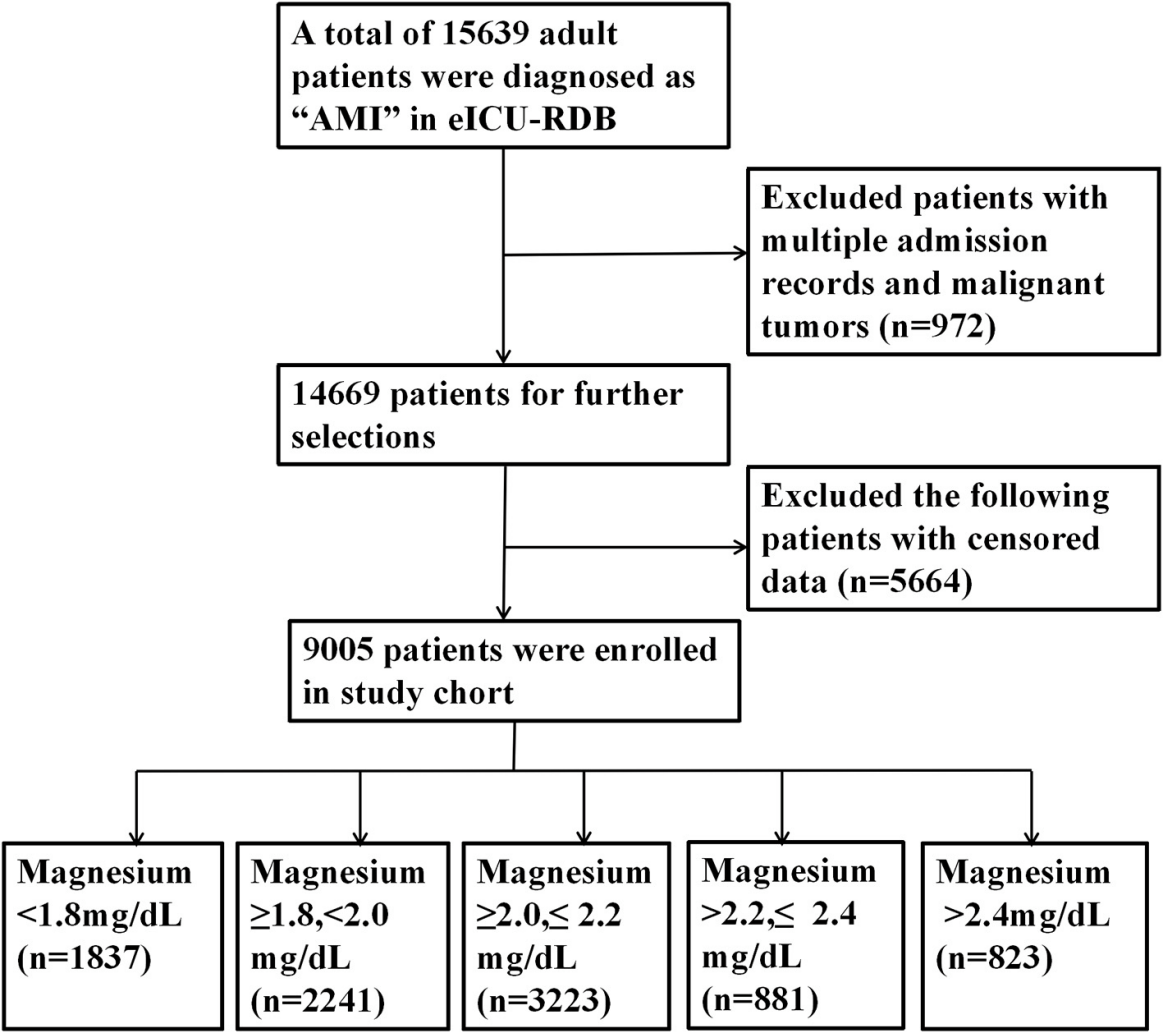
Flowchart outlining patient selection and grouping process.

We included adult patients with AMI on the basis of the ninth revision of the *International Classification of Diseases* (ICD9) adopted in eICU-CRD. Patients were excluded because of the following standards: (1) patients with multiple admission records and malignant tumors; (2) no serum magnesium data; and (3) missing >5% individual data. The complete inclusion and exclusions processes are listed in Figure 1.

### Data Stratification

Similar to previous studies, data extraction was conducted with PostgreSQL tools (version 9.6) through Structured Query Language (SQL). Demographic data, comorbidities, vital signs, laboratory variables, use of vasoactive drugs, magnesium supplementation, and other data were obtained from eICU-CRD. The laboratory variables included white blood cell (WBC), platelet (PLT), red blood cell (RBC), hemoglobin (Hb), blood urea nitrogen (BUN), creatinine (Cr), glucose, serum potassium, sodium, bicarbonate, total calcium, and serum magnesium. Comorbidities were also extracted, including hypertension, congestive heart failure (CHF), diabetes, peripheral vascular disease (PVD), prior myocardial infarction (MI), atrial fibrillation, valvular disease, cardiogenic shock, cardiac arrest, and hypercholesterolemia (HC). The vital signs included heart rate (HR), systolic blood pressure (SBP), diastolic blood pressure (DBP), and mean blood pressure (MBP). Used drugs included norepinephrine, dopamine, epinephrine, and magnesium supplement. Other demographic information included age, gender ethnicity, and hospital mortality. Only the data for the first ICU admission of each patient were used, and baseline data were extracted within 24 h after ICU admission.

### Serum Magnesium Measurement

All values of serum magnesium levels were extracted from the lab table in eICU-CRD, which were collected during routine care, and are interfaced with eCareManager and archived in the database. All laboratory measurements were performed at the local accredited hospital laboratory. Then, each hospital has had its local laboratory measurements mapped to standard concepts. As a result, the results of serum magnesium levels are well-harmonized and comparable. The database has extensive documentation of how data elements are measured as well as their mapping methods (12).

### Statistical Analysis

All the baseline data of eligible items were stratified according to admission serum magnesium levels (<1.8 mg/dl; ≥1.8, <2.0 mg/dl; ≥2.0, ≤2.2 mg/dl; >2.2, ≤2.4 mg/dl; and >2.4 mg/dl), and mean ± standard deviation (SD) or medians with interquartile range (IQR) were used to present continuous index. Categorical variables were presented as number and percentage, and the chi-squared test was used to examine the difference. Cox regression analysis of 30-day in-hospital mortality was performed according to five serum magnesium groups to explore the association between in-hospital mortality and baseline covariates. The third group was set as a reference, and odds ratios (ORs) or hazard ratios (HRs) and 95% confidence intervals (CIs) were used to present all the results.

Three multivariate models for hospital mortality were used to examine clinical interpretation of univariate logistic and Cox analysis results. In model 1, covariates were adjusted for age, gender, and ethnicity. In model 2, we adjusted for age, ethnicity, gender, hypertension, CHF, diabetes, PVD, prior MI, atrial fibrillation, valvular disease, cardiogenic shock, cardiac arrest, and HC. In model 3, we further adjusted for age, ethnicity, gender, hypertension, CHF, diabetes, PVD, prior MI, atrial fibrillation, valvular disease, cardiogenic shock, cardiac arrest, HC, HR, SBP, DBP, MBP, norepinephrine, dopamine, epinephrine, magnesium supplementation, WBC, PLT, RBC, Hb, BUN, Cr, glucose, serum potassium, sodium, bicarbonate, and total calcium. Variables on the basis of the possible association were included as potential confounding factors, and these confounding factors basing on a change in effect estimate of >10% were used to construct an adjusted model. Moreover, we compared the survival rates within five serum magnesium groups using log-rank tests, and we present the results as Kaplan-Meier (K-M) curves.

We also conducted subgroup analyses to evaluate the relationship between the admission serum magnesium and in-hospital mortality in different subgroups, including age, ethnicity, gender, hypertension, CHF, diabetes, PVD, prior MI, atrial fibrillation, valvular disease, cardiogenic shock, cardiac arrest, HC, HR, SBP, DBP, MBP, norepinephrine, dopamine, epinephrine, WBC, PLT, RBC, Hb, BUN, Cr, glucose, serum potassium, sodium, bicarbonate, and total calcium. Continuous variables should be converted to dichotomous groups based on the median (< median and ≥median). The analysis was mainly performed by EmpowerStats software (http://www.empowerstats.com/cn/, X&Y solutions, Inc., Boston, MA). All probability values were two-sided, and a *P*-value for interaction <0.05 was considered statistically significant.

## Results

### Patient Characteristics

In this study, 9,005 eligible cases were included. The baseline characteristics of all patients were stratified according to serum magnesium tertiles (Table 1). Thereinto, 1,837 patients were assigned to group 1 (<1.8 mg/dl), 2,241 patients to group 2 (≥1.8, <2.0 mg/dl), 3,223 patients to group 3 (≥2.0, ≤2.2 mg/dl), 881 patients to group 4 (>2.2, ≤2.4 mg/dl), and 823 patients to group 5 (>2.4 mg/dl). Table 1 shows the complicated relationships between baseline characteristics of the AMI patients based on five magnesium strata. Patients with higher serum magnesium levels were more likely to be older and male, with comorbidities of hypertension, CHF, diabetes, atrial fibrillation, valvular disease, cardiogenic shock, and cardiac arrest. Moreover, patients with higher serum magnesium levels had more use of vasoactive drugs and less supplement of magnesium.

**Table 1:** Characteristics of the included patients according to serum magnesium levels.

**Characteristics**	**Serum magnesium levels (mg/dl)**					
	** <1.8 (*n* = 1,837)**	**≥1.8; <2.0 (*n* = 2,241)**	**≥2.0; ≤2.2 (*n* = 3,223)**	**>2.2; ≤2.4 (*n* = 881)**	**>2.4 (*n* = 823)**	***P-*value**
Age, years	66.87 ± 13.08	66.67 ± 13.42	66.69 ± 13.41	67.27 ± 13.28	67.89 ± 13.01	0.153
Gender, *n* (%)						<0.001
Female	826 (44.96%)	842 (37.57%)	1,049 (32.55%)	307 (34.85%)	302 (36.70%)	
Male	1,011 (55.04%)	1,399 (62.43%)	2,174 (67.45%)	574 (65.15%)	521 (63.30%)	
Ethnicity, *n* (%)						<0.001
African American	209 (11.38%)	181 (8.08%)	257 (7.97%)	89 (10.10%)	70 (8.51%)	
Asian	27 (1.47%)	28 (1.25%)	68 (2.11%)	14 (1.59%)	24 (2.92%)	
Caucasian	1,455 (79.21%)	1,802 (80.41%)	2,583 (80.14%)	693 (78.66%)	652 (79.22%)	
Hispanic	70 (3.81%)	99 (4.42%)	126 (3.91%)	35 (3.97%)	43 (5.22%)	
Native American	7 (0.38%)	5 (0.22%)	12 (0.37%)	5 (0.57%)	2 (0.24%)	
Others	69 (3.76%)	126 (5.62%)	177 (5.49%)	45 (5.11%)	32 (3.89%)	
Comorbidities, *n* (%)						
Hypertension	1,313 (71.48%)	1,471 (65.64%)	2,061 (63.95%)	617 (70.03%)	546 (66.34%)	<0.001
CHF	746 (23.15%)	448 (24.39%)	488 (21.78%)	255 (28.94%)	230 (27.95%)	<0.001
Diabetes	499 (27.16%)	494 (22.04%)	687 (21.32%)	224 (25.43%)	204 (24.79%)	<0.001
PVD	124 (6.75%)	130 (5.80%)	176 (5.46%)	49 (5.56%)	54 (6.56%)	0.359
Prior MI	394 (21.45%)	481 (21.46%)	700 (21.72%)	176 (19.98%)	166 (20.17%)	0.746
Atrial fibrillation	283 (15.41%)	305 (13.61%)	470 (14.58%)	155 (17.59%)	149 (18.10%)	0.005
Valvular disease	61 (3.32%)	97 (4.33%)	133 (4.13%)	47 (5.33%)	53 (6.44%)	0.003
Cardiogenic shock	111 (6.04%)	125 (5.58%)	200 (6.21%)	69 (7.83%)	84 (10.21%)	<0.001
Cardiac arrest	123 (6.70%)	147 (6.56%)	240 (7.45%)	81 (9.19%)	117 (14.22%)	<0.001
HC	179 (9.74%)	164 (7.32%)	266 (8.25%)	78 (8.85%)	68 (8.26%)	0.089
Vital signs						
HR (beats/min)	86.18 ± 20.23	83.44 ± 19.68	83.39 ± 19.76	86.02 ± 19.07	86.65 ± 19.47	<0.001
SBP (mmHg)	123.58 ± 26.38	124.11 ± 25.44	124.92 ± 24.74	124.36 ± 26.67	120.56 ± 26.05	<0.001
DBP (mmHg)	70.59 ± 17.07	71.49 ± 17.21	71.84 ± 17.00	69.93 ± 18.11	68.38 ± 17.05	<0.001
MBP (mmHg)	88.26 ± 18.41	89.03 ± 18.29	89.53 ± 17.82	88.07 ± 19.11	85.77 ± 18.08	<0.001
Norepinephrine	175 (9.53%)	198 (8.84%)	348 (10.80%)	135 (15.32%)	143 (17.38%)	<0.001
Dopamine	15 (0.82%)	10 (0.45%)	26 (0.81%)	9 (1.02%)	15 (1.82%)	0.007
Epinephrine	34 (1.85%)	35 (1.56%)	72 (2.23%)	31 (3.52%)	33 (4.01%)	<0.001
Mg supplement	284 (15.46%)	301 (13.43%)	350 (10.86%)	64 (7.26%)	31 (3.52%)	<0.001
WBC (K/μl)	13.59 ± 6.70	13.29 ± 8.26	13.43 ± 7.07	15.14 ± 9.36	15.72 ± 10.81	<0.001
PLT (K/μl)	237.06 ± 94.74	233.79 ± 88.20	237.59 ± 89.49	235.16 ± 93.46	225.72 ± 93.23	0.014
RBC (m/μl)	3.92 ± 0.78	4.00 ± 0.76	4.01 ± 0.79	3.86 ± 0.80	3.72 ± 0.77	<0.001
Hb (g/dl)	12.79 ± 2.30	13.00 ± 2.24	13.14 ± 2.31	12.83 ± 2.40	12.69 ± 2.45	<0.001
BUN (mg/dl)	27.46 ± 19.41	26.31 ± 20.24	27.18 ± 18.95	34.40 ± 26.85	37.62 ± 28.16	<0.001
Cr (mg/dl)	1.67 ± 1.53	1.61 ± 1.59	1.60 ± 1.52	2.11 ± 2.45	2.13 ± 1.89	<0.001
Glucose (mg/dl)	201.43 ± 120.70	185.06 ± 101.10	183.74 ± 110.42	204.48 ± 119.24	222.97 ± 156.92	<0.001
Potassium (mmol/L)	4.42 ± 0.72	4.40 ± 0.69	4.45 ± 0.68	4.62 ± 0.79	4.74 ± 0.86	<0.001
Sodium (mmol/L)	139.08 ± 4.51	139.45 ± 4.23	139.53 ± 4.37	139.95 ± 4.88	140.32 ± 5.19	<0.001
Bicarbonate (mmol/L)	24.89 ± 4.04	25.52 ± 3.85	25.71 ± 3.89	25.47 ± 4.40	24.87 ± 4.41	<0.001
Calcium (mg/dl)	8.51 ± 0.79	8.56 ± 0.71	8.58 ± 0.74	8.49 ± 0.85	8.40 ± 0.86	<0.001
Hospital mortality	180 (9.80%)	164 (7.32%)	297 (9.22%)	155 (17.59%)	161 (19.56%)	<0.001

*CHF, congestive heart failure; PVD, peripheral vascular disease; prior MI, prior myocardial infarction; HC, hypercholesterolemia; HR, heart rate; SBP, systolic blood pressure; DBP, diastolic blood pressure; MBP, mean blood pressure; WBC, white blood cell; PLT, platelet; RBC, red blood cell; Hb, Hemoglobin; BUN, blood urea nitrogen; Cr, creatinine*.

### Association Between Serum Magnesium Levels and Hospital Mortality

**Figure 2:**
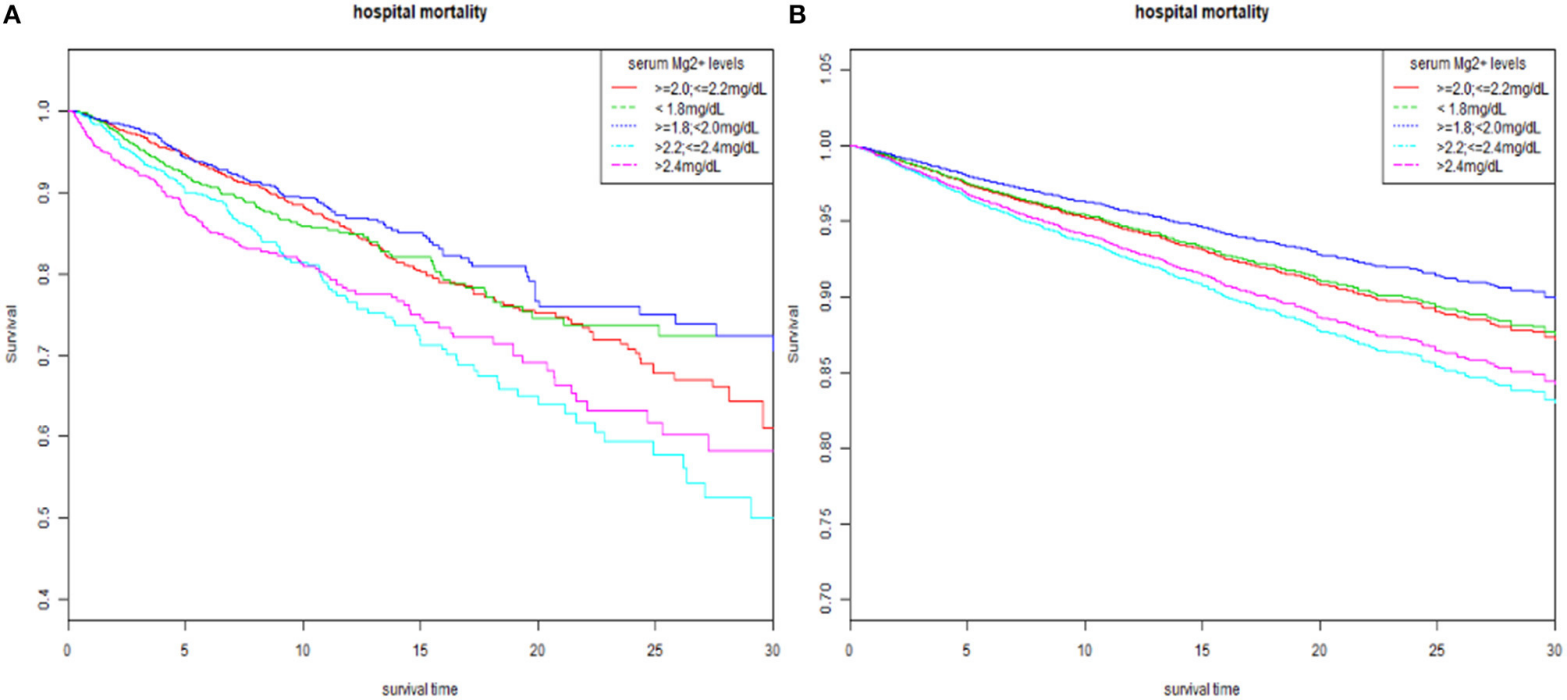
The K-M survival curve of five strata of serum magnesium. **(A)** Unadjusted K-M survival curve. **(B)** Adjusted for age, ethnicity, gender, hypertension, CHF, diabetes, PVD, prior MI, atrial fibrillation, valvular disease, cardiogenic shock, cardiac arrest, HC, HR, SBP, DBP, MBP, norepinephrine, dopamine, epinephrine, Mg supplement, WBC, PLT, RBC, Hb, BUN, Cr, glucose, serum potassium, sodium, bicarbonate, and total calcium. K-M, Kaplan-Meier; CHF, congestive heart failure; PVD, peripheral vascular disease; MI, myocardial infarction; HC, hypercholesterolemia; HR, heart rate; SBP, systolic blood pressure; DBP, diastolic blood pressure; MBP, mean blood pressure; WBC, white blood cell; PLT, platelet; RBC, red blood cell; Hb, hemoglobin; BUN, blood urea nitrogen; Cr, creatinine.

We analyzed serum magnesium levels stratified into five strata to explore whether serum magnesium levels were independently associated with hospital mortality via multivariate logistic regression analysis (Table 2). In model 1, adjusted for age, ethnicity, and gender, compared with the referent group (≥2.0, ≤2.2 mg/dl), high magnesium levels (group 4 and group 5) were significant predictors of all-cause hospital mortality in patients with AMI (OR, 2.11 and 2.33, respectively). In model 2, after age, ethnicity, gender, hypertension, CHF, diabetes, PVD, prior MI, atrial fibrillation, valvular disease, cardiogenic shock, cardiac arrest, and HC were adjusted, high magnesium levels remained a significant predictor of hospital mortality in AMI patients (OR, 2.02 and 1.97). Finally, In model 3, after age, ethnicity, gender, hypertension, CHF, diabetes, PVD, prior MI, atrial fibrillation, valvular disease, cardiogenic shock, cardiac arrest, HC, HR, SBP, DBP, MBP, norepinephrine, dopamine, epinephrine, WBC, PLT, RBC, Hb, BUN, Cr, glucose, serum potassium, sodium, bicarbonate, and total calcium were adjusted, higher magnesium levels continued to be a significant predictor of hospital mortality (OR, 1.63 vs. 1.39). The results from Cox regression to 30-day in-hospital mortality and K-M curve also presented similar results (Table 3 and Figure 2).

**Table 2:** ORs (95% CIs) for mortality across groups of serum magnesium.

**Serum Mg (mg/dl)**		** <1.8**	**≥1.8; <2.0**	**≥2.0, ≤2.2**	**>2.2; ≤2.4**	**>2.4**
Non- adjusted	OR (95% CIs)	1.07 (0.88, 1.30)	0.78 (0.64, 0.95)	1.0 (ref)	2.10 (1.70, 2.60)	2.40 (1.94, 2.96)
	*P*-value	0.495	0.013		<0.001	<0.001
Model 1	OR (95% CIs)	1.04 (0.86, 1.27)	0.77 (0.63, 0.94)	1.0 (ref)	2.11 (1.70, 2.61)	2.33 (1.89, 2.89)
	*P*-value	0.679	0.009		<0.001	<0.001
Model 2	OR (95% CIs)	1.07 (0.87, 1.31)	0.77 (0.62, 0.95)	1.0 (ref)	2.02 (1.61, 2.53)	1.97 (1.57, 2.47)
	*P*-value	0.542	0.014		<0.001	<0.001
Model 3	OR (95% CIs)	0.89 (0.72, 1.11)	0.72 (0.58, 0.89)	1.0 (ref)	1.63 (1.28, 2.06)	1.39 (1.09, 1.77)
	*P*-value	0.305	0.003		<0.001	0.007

*Models were derived from logistic regression models. Non-adjusted model adjust for: none. Adjust model 1 adjust for: age, ethnicity, and gender. Adjust model 2 adjust for: age, ethnicity, gender, hypertension, CHF, diabetes, PVD, prior MI, atrial fibrillation, valvular disease, cardiogenic shock, cardiac arrest, and HC. Adjust model 3 adjust for: age, ethnicity, gender, hypertension, CHF, diabetes, PVD, prior MI, atrial fibrillation, valvular disease, cardiogenic shock, cardiac arrest, HC, HR, SBP, DBP, MBP, norepinephrine, dopamine, epinephrine, Mg supplement, WBC, PLT, RBC, Hb, BUN, Cr, glucose, serum potassium, sodium, bicarbonate, and total calcium*.

*CHF, congestive heart failure; PVD, peripheral vascular disease; MI, myocardial infarction; HC, hypercholesterolemia; HR, heart rate; SBP, systolic blood pressure; DBP, diastolic blood pressure; MBP, mean blood pressure; WBC, white blood cell; PLT, platelet; RBC, red blood cell; Hb, hemoglobin; BUN, blood urea nitrogen; Cr, creatinine*.

**Table 3:** HRs (95% CIs) for mortality across groups of serum magnesium.

**Serum Mg (mg/dl)**		** <1.8**	**≥1.8; <2.0**	**≥2.0; ≤2.2**	**>2.2; ≤2.4**	**>2.4**
Non- adjusted	HR (95% CIs)	1.09 (0.91, 1.32)	0.84 (0.69, 1.01)	1.0 (ref)	1.60 (1.32, 1.94)	1.61 (1.33, 1.95)
	*P*-value	0.342	0.067		<0.001	<0.001
Model 1	HR (95% CIs)	1.11 (0.92, 1.34)	0.83 (0.69, 1.01)	1.0 (ref)	1.65 (1.36, 2.00)	1.64 (1.35, 1.99)
	*P*-value	0.279	0.059		<0.001	<0.001
Model 2	HR (95% CIs)	1.16 (0.96, 1.40)	0.83 (0.69, 1.01)	1.0 (ref)	1.60 (1.31, 1.94)	1.46 (1.20, 1.77)
	*P*-value	0.122	0.057		<0.001	<0.001
Model 3	HR (95% CIs)	0.97 (0.80, 1.17)	0.78 (0.64, 0.94)	1.0 (ref)	1.36 (1.12, 1.66)	1.25 (1.03, 1.52)
	*P*-value	0.745	0.009		0.002	0.026

*Models were derived from Cox regression models. Non-adjusted model adjust for: none. Adjust model 1 adjust for: age, ethnicity, and gender. Adjust model 2 adjust for: age, ethnicity, gender, hypertension, CHF, diabetes, PVD, prior MI, atrial fibrillation, valvular disease, cardiogenic shock, cardiac arrest and HC. Adjust model 3 adjust for: age, ethnicity, gender, hypertension, CHF, diabetes, PVD, prior MI, atrial fibrillation, valvular disease, cardiogenic shock, cardiac arrest, HC, HR, SBP, DBP, MBP, norepinephrine, dopamine, epinephrine, Mg supplement, WBC, PLT, RBC, Hb, BUN, Cr, glucose, serum potassium, sodium, bicarbonate, and total calcium*.

*CHF, congestive heart failure; PVD, peripheral vascular disease; MI, myocardial infarction; HC, hypercholesterolemia; HR, heart rate; SBP, systolic blood pressure; DBP, diastolic blood pressure; MBP, mean blood pressure; WBC, white blood cell; PLT, platelet; RBC, red blood cell; Hb, hemoglobin; BUN, blood urea nitrogen; Cr, creatinine*.

### Subgroup Analyses

We conducted subgroup analyses subsequently to access the relationship between the serum magnesium levels at ICU admission and in-hospital mortality in different subgroups (Table 4). The result showed that patients with younger age (<67 years), combined with cardiac arrest and without epinephrine use, had an increased hospital mortality risk with high magnesium levels. Moreover, patients with high magnesium also showed a higher risk of mortality in higher Hb (>13.1 g/dl); RBC (>3.96 m/all) and total calcium (≥8.6 mg/dl) subgroup; and lower BUN (<21 mg/dl) and potassium (<4.3 mmol/L) subgroup. Furthermore, the results of the subgroup analyses to Cox regression also yielded similar results (Supplementary Table 1).

**Table 4:** Subgroup analyses of the associations between serum magnesium levels and in-hospital mortality in logistic regression model.

**Variables**	**Serum Mg levels (mmol/L)**					**Interaction *P*-value**
	** <1.8**	**≥1.8; <2.0**	**≥2.0, ≤2.2**	**>2.2; ≤2.4**	**>2.4**	
Gender						0.357
Female	1.23 (0.92, 1.64)	0.84 (0.62, 1.15)	Ref	2.45 (1.76, 3.43)	2.32 (1.65, 3.26)	
Male	0.87 (0.66, 1.15)	0.72 (0.55, 0.93)	Ref	1.89 (1.44, 2.48)	2.42 (1.85, 3.15)	
Age, years						0.043
<67	1.17 (0.81, 1.69)	0.84 (0.62, 1.15)	Ref	2.45 (1.76, 3.43)	2.32 (1.65, 3.26)	
≥67	1.01 (0.80, 1.28)	0.73 (0.57, 0.92)	Ref	2.05 (1.59, 2.65)	1.85 (1.42, 2.39)	
Ethnicity						0.212
African American	1.81 (0.84, 3.88)	0.70 (0.26, 1.90)	Ref	0.40 (0.05, 3.37)	4.22 (1.81, 9.88)	
Asian	1.18 (0.37, 3.78)	0.19 (0.02, 1.56)	Ref	0.40 (0.05, 3.37)	0.47 (0.10, 2.30)	
Caucasian	1.04 (0.83, 1.29)	0.78 (0.62, 0.97)	Ref	2.11 (1.67, 2.67)	2.46 (1.95, 3.10)	
Hispanic	1.80 (0.77, 4.19)	1.31 (0.58, 2.98)	Ref	2.58 (0.97, 6.83)	1.41 (0.50, 3.97)	
Native American	/	/	Ref	2.75 (0.14, 55.17)	/	
Others	0.53 (0.15, 1.90)	0.79 (0.32, 1.94)	Ref	1.46 (0.50, 4.28)	3.26 (1.20, 8.86)	
Hypertension						0.497
No	0.89 (0.61, 1.29)	0.86 (0.62, 1.20)	Ref	1.82 (1.23, 2.69)	2.35 (1.64, 3.38)	
Yes	1.14 (0.90, 1.43)	0.74 (0.57, 0.94)	Ref	2.22 (1.73, 2.85)	2.41 (1.87, 3.12)	
CHF						0.285
No	1.03 (0.81, 1.31)	0.76 (0.60, 0.98)	Ref	1.96 (1.50, 2.56)	2.67 (2.06, 3.44)	
Yes	1.12 (0.81, 1.55)	0.83 (0.59, 1.17)	Ref	2.15 (1.52, 3.04)	1.81 (1.25, 2.62)	
Diabetes						0.070
No	1.22 (0.98, 1.54)	0.88 (0.70, 1.10)	Ref	2.17 (1.69, 2.78)	2.68 (2.10, 3.41)	
Yes	0.71 (0.49, 1.04)	0.53 (0.35, 0.80)	Ref	1.86 (1.25, 2.76)	1.70 (1.12, 2.57)	
PVD						0.709
No	1.08 (0.88, 1.32)	0.80 (0.65, 0.98)	Ref	2.13 (1.71, 2.65)	2.48 (2.00, 3.09)	
Yes	0.92 (0.47, 1.77)	0.53 (0.25, 1.12)	Ref	1.87 (0.86, 4.05)	1.48 (0.68, 3.23)	
Prior MI						0.741
No	1.11 (0.89, 1.38)	0.80 (0.64, 1.00)	Ref	2.10 (1.66, 2.65)	2.54 (2.02, 3.20)	
Yes	0.91 (0.58, 1.43)	0.68 (0.43, 1.08)	Ref	2.10 (1.30, 3.39)	1.80 (1.09, 2.99)	
Atrial fibrillation						0.351
No	1.14 (0.91, 1.43)	0.86 (0.68, 1.07)	Ref	2.11 (1.65, 2.70)	2.58 (2.03, 3.29)	
Yes	0.85 (0.57, 1.27)	0.57 (0.37, 0.88)	Ref	1.94 (1.27, 2.95)	1.74 (1.13, 2.68)	
Valvular disease						0.484
No	1.07 (0.88, 1.31)	0.78 (0.63, 0.95)	Ref	1.99 (1.60, 2.48)	2.39 (1.92, 2.97)	
Yes	1.18 (0.49, 2.82)	0.78 (0.34, 1.80)	Ref	3.87 (1.77, 8.46)	2.22 (0.99, 4.97)	
Cardiogenic shock						0.010
No	1.08 (0.87, 1.34)	0.79 (0.63, 0.98)	Ref	2.22 (1.76, 2.80)	2.65 (2.11, 3.33)	
Yes	1.07 (0.65, 1.76)	0.77 (0.46, 1.26)	Ref	1.43 (0.81, 2.53)	1.00 (0.57, 1.73)	
Cardiac arrest						0.225
No	0.98 (0.78, 1.22)	0.74 (0.59, 0.93)	Ref	2.01 (1.59, 2.55)	2.03 (1.58, 2.60)	
Yes	1.80 (1.13, 2.86)	1.05 (0.66, 1.67)	Ref	2.48 (1.47, 4.19)	2.67 (1.68, 4.24)	
HC						0.243
No	1.12 (0.92, 1.37)	0.77 (0.63, 0.95)	Ref	2.05 (1.65, 2.56)	2.41 (1.94, 2.99)	
Yes	0.53 (0.22, 1.29)	0.84 (0.38, 1.86)	Ref	2.84 (1.35, 5.98)	2.24 (0.99, 5.08)	
Norepinephrine						0.490
No	1.11 (0.89, 1.39)	0.77 (0.62, 0.98)	Ref	2.02 (1.57, 2.59)	2.43 (1.90, 3.10)	
Yes	1.04 (0.68, 1.60)	0.91 (0.60, 1.39)	Ref	1.94 (1.26, 2.98)	1.72 (1.12, 2.63)	
Dopamine						0.054
No	1.07 (0.88, 1.31)	0.79 (0.64, 0.96)	Ref	2.11 (1.71, 2.61)	2.47 (2.00, 3.04)	
Yes	0.85 (0.14, 5.28)	/	Ref	1.57 (0.24, 10.49)	/	
Epinephrine						0.016
No	1.06 (0.87, 1.30)	0.79 (0.65, 0.97)	Ref	2.14 (1.72, 2.65)	2.53 (2.05, 3.14)	
Yes	1.46 (0.58, 3.67)	0.58 (0.19, 1.75)	Ref	1.22 (0.46, 3.24)	0.48 (0.15, 1.58)	
Mg supplement						0.072
No	1.07 (0.89, 1.29)	0.79 (0.69, 1.24)	Ref	2.06 (1.58, 2.73)	2.22 (1.72, 2.99)	
Yes	1.02 (0.69, 1.62)	0.71 (0.43, 0.85)	Ref	2.15 (1.48, 2.95)	2.38 (1.69, 3.48)	
HR (beats/min)						0.066
<83	0.85 (0.60, 1.21)	0.72 (0.51, 1.00)	Ref	2.41 (1.72, 3.38)	2.87 (2.05, 4.02)	
≥83	1.10 (0.87, 1.40)	0.81 (0.63, 1.04)	Ref	1.83 (1.39, 2.40)	2.01 (1.53, 2.63)	
SBP, mmHg						0.283
<122	1.07 (0.83, 1.38)	0.89 (0.69, 1.14)	Ref	2.18 (1.65, 2.88)	2.18 (1.67, 2.85)	
≥122	1.04 (0.77, 1.42)	0.61 (0.43, 0.85)	Ref	2.02 (1.46, 2.80)	2.52 (1.80, 3.54)	
DBP, mmHg			Ref			0.703
<70	1.00 (0.77, 1.29)	0.83 (0.65, 1.08)		2.06 (1.57, 2.72)	2.28 (1.74, 2.99)	
≥70	1.13 (0.84, 1.52)	0.67 (0.49, 0.93)	Ref	2.05 (1.47, 2.85)	2.34 (1.67, 3.28)	
MBP, mmHg						0.842
<88	1.07 (0.84, 1.37)	0.84 (0.65, 1.07)	Ref	2.10 (1.60, 2.75)	2.22 (1.71, 2.89)	
≥88	1.02 (0.74, 1.40)	0.66 (0.48, 0.93)	Ref	2.06 (1.47, 2.88)	2.38 (1.67, 3.38)	
WBC, K/μl						0.266
<12.3	1.22 (0.85, 1.75)	[n] (0.70, 1.44)	Ref	2.72 (1.84, 4.01)	2.83 (1.89, 4.25)	
≥12.3	1.01 (0.80, 1.28)	0.70 (0.55, 0.89)	Ref	1.73 (1.34, 2.24)	1.97 (1.54, 2.53)	
Platelet, K/μl						0.837
<223	1.07 (0.81, 1.42)	0.72 (0.54, 0.96)	Ref	2.08 (1.54, 2.81)	2.54 (1.91, 3.38)	
≥223	1.07 (0.81, 1.40)	0.84 (0.64, 1.10)	Ref	2.13 (1.59, 2.86)	2.21 (1.62, 3.03)	
Hb, g/dl						0.002
<13.1	0.90 (0.70, 1.15)	0.79 (0.62, 1.02)	Ref	1.85 (1.41, 2.43)	1.65 (1.25, 2.19)	
≥13.1	1.25 (0.91, 1.70)	0.70 (0.50, 0.98)	Ref	2.32 (1.66, 3.24)	3.60 (2.62, 4.95)	
RBC, K/μl						<0.001
<3.96	0.87 (0.68, 1.11)	0.83 (0.65, 1.05)	Ref	1.70 (1.31, 2.20)	1.41 (1.08, 1.84)	
≥3.96	1.40 (1.01, 1.95)	0.67 (0.46, 0.96)	Ref	2.60 (1.80, 3.75)	4.95 (3.49, 7.02)	
BUN, mg/dl						<0.001
<21	1.80 (1.16, 2.81)	1.14 (0.72, 1.80)	Ref	2.34 (1.35, 4.05)	5.47 (3.39, 8.84)	
≥21	0.92 (0.74, 1.15)	0.76 (0.60, 0.95)	Ref	1.81 (1.43, 2.29)	1.60 (1.26, 2.03)	
Cr, mg/dl						0.286
<1.17	1.26 (0.85, 1.85)	0.73 (0.48, 1.11)	Ref	1.75 (1.07, 2.85)	2.84 (1.80, 4.49)	
≥1.17	0.98 (0.78, 1.24)	0.81 (0.64, 1.02)	Ref	1.85 (1.45, 2.36)	1.82 (1.43, 2.31)	
Glucose, mg/dl						0.183
<158	1.24 (0.88, 1.75)	0.74 (0.52, 1.06)	Ref	2.67 (1.85, 3.85)	2.37 (1.60, 3.51)	
≥158	0.91 (0.72, 1.16)	0.77 (0.61, 0.99)	Ref	1.66 (1.28, 2.16)	2.07 (1.61, 2.67)	
Potassium, mmol/L						0.028
<4.3	1.22 (0.87, 1.70)	0.73 (0.51, 1.05)	Ref	2.21 (1.48, 3.29)	3.65 (2.50, 5.33)	
≥4.3	1.03 (0.81, 1.31)	0.82 (0.64, 1.04)	Ref	1.92 (1.50, 2.47)	1.85 (1.44, 2.38)	
Sodium, mmol/L						0.881
<140	1.01 (0.76, 1.35)	0.70 (0.52, 0.94)	Ref	2.09 (1.54, 2.85)	2.27 (1.64, 3.14)	
≥140	1.14 (0.87, 1.49)	0.85 (0.65, 1.11)	Ref	2.11 (1.58, 2.82)	2.45 (1.86, 3.23)	
Bicarbonate, mmol/L						0.635
<25	0.90 (0.70, 1.17)	0.73 (0.56, 0.95)	Ref	1.78 (1.33, 2.39)	2.03 (1.54, 2.69)	
≥25	1.10 (0.80, 1.50)	0.80 (0.59, 1.08)	Ref	2.42 (1.77, 3.29)	2.55 (1.85, 3.53)	
Calcium, mg/dl						0.044
<8.6	0.94 (0.74, 1.20)	0.73 (0.57, 0.94)	Ref	1.75 (1.34, 2.29)	1.74 (1.34, 2.27)	
≥8.6	1.19 (0.85, 1.66)	0.82 (0.58, 1.14)	Ref	2.56 (1.81, 3.61)	3.37 (2.38, 4.78)	

*CHF, congestive heart failure; PVD, peripheral vascular disease; MI, myocardial infarction; HC, hypercholesterolemia; HR, heart rate; SBP, systolic blood pressure; DBP, diastolic blood pressure; MBP, mean blood pressure; WBC, white blood cell; PLT, platelet; RBC, red blood cell; Hb, hemoglobin; BUN, blood urea nitrogen; Cr, creatinine*.

## Discussion

AMI is the leading cause of global deaths over the past 15 years. Despite a decreasing trend of AMI mortality in developed countries owing to an advance in emergency revascularization, the mortality of AMI in developing countries still increased year by year (16), and most of it occurs more at the early stage of ICU patients since the high incidence of malignant arrhythmias (5, 17). However, a simple and early-stage biomarker for in-hospital mortality is still lacking. In this situation, the exploration of the early-stage biomarker to ICU patients is critical for indicating the risk of in-hospital death.

Generally, biochemical variables in peripheral blood can be determined to reflect the subtle physical changes and prognosis. Hence, many studies focused on exploring serum biomarkers to the mortality of AMI, including C-reactive protein (18), albumin (19), and neutrophil–lymphocyte ratio (20). Magnesium, especially intracellular magnesium, is an essential mineral for the cardiovascular system. Although serum magnesium levels may not reflect the expression of intracellular magnesium, the turbulence of serum magnesium can also lead to cardiovascular dysfunction (21). More importantly, serum magnesium is an easily detected, cheap laboratory marker with a short turnaround time (TAT), which may promote its application in clinical practice (22). Growing evidence supports an increased cardiovascular disease (CVD) risk with low dietary magnesium intake and a benefit for magnesium supplementation in the treatment of AMI (23). The mechanisms of intracellular magnesium regulating cardiac physiology were widely studied; therefore, many small sample-size and single-center studies provided contradictory conclusions about the relationship between serum magnesium and AMI mortality (24, 25). Most researches indicated the inverse correlation between admission serum magnesium levels and the risk of mortality (26–28). However, other studies showed that admission serum magnesium level could not predict the hospital outcome of patients with AMI (25, 29). Hence, the association between the admission serum magnesium with hospital mortality needs more solid clinical validations.

In our study, in order to evaluate the association between the admission serum magnesium with hospital mortality in ICU patients with AMI, we included 9,005 patients. In three kinds of multivariate analysis, admission serum higher magnesium levels (≥2.2 mg/dl) was recognized as a significant predictor of in-hospital mortality. The result of the subgroup analyses showed that patients with younger age (<67 years), combined with cardiac arrest and without epinephrine use, showed an increased hospital mortality risk with high serum magnesium levels. Moreover, patients with high magnesium also showed higher risk of mortality in higher Hb, RBC, and total calcium subgroup, and lower BUN and potassium subgroup. This study is the first to confirm the association of high-normal serum magnesium and in-hospital mortality through multicenter EHR data.

Previous clinical studies mainly focused on mortality and hypomagnesemia, which highly occur in AMI patients. In our study, serum low-magnesium-level and high-magnesium-level groups had a higher risk of in-hospital mortality (group 1, 9.80%; group 2, 7.32%; group 3, 9.22%; group 4, 17.59%; and group 5, 19.56%). However, in the logistic and Cox regression model and models adjusted by other covariates, higher serum magnesium was a significant predictor of in-hospital mortality of AMI. However, guidelines recommend magnesium supplementation regularly to maintain levels >2.0 mg/dl during AMI (30), because early-stage epidemiological investigation showed that serum magnesium is associated inversely with risk factors for coronary heart disease. Other evidence from clinical, ecologic, and autopsy researches presented that high serum magnesium could protect against sudden cardiac death (31). However, these studies were mainly conducted in the community population or all patients from the clinics and hospitals.

Our study indicated that higher admission magnesium levels, especially high-normal magnesium levels (>2.2, ≤2.4 mg/dl), were highly correlated to in-hospital mortality, which may indicate the particularity of the ICU patients with AMI. In ICU patients, obvious electrolyte disturbance such as hypermagnesemia and hypomagnesemia could always be corrected during careful monitoring. Therefore, high-normal serum magnesium with a higher risk of mortality would not be attached with enough importance. Additionally, different strategies to magnesium supplementation in different hospitals could also explain inverse results in single-center research. Our findings indicated that the recently recommended serum magnesium level may be not suitable for ICU patients with AMI. Moreover, patients with admission high-normal magnesium levels should be also carefully monitored in the same way as patients with hypermagnesemia.

Actually, an increasing number of clinical studies questioned the benefit of magnesium supplementation and the secure range of serum magnesium for AMI patients. A study from the *Journal of the American College of Cardiology* showed that the optimal range of magnesium in patients with AMI should be lower than what is currently recommended by AMI guidelines (26). Moreover, the ISIS-4 trial (Fourth International Study of Infarct Survival) included a large group of 58,050 patients with suspected AMI but did not show a positive effect of magnesium supplementation (32). In this situation, clinical magnesium supplementation should done more carefully with close monitoring of serum magnesium.

The association between high magnesium levels and higher in-hospital mortality in AMI patients might be explained as follows: firstly, in cardiac myocytes, magnesium ions were reported to compete with calcium ion-activating and inactivating sites on the type II isoform ryanodine receptor channels; thus, high serum magnesium could cause damage to both cardiac contraction and relaxation (33). Secondly, high serum magnesium may impair the release of acetylcholine and reduce motor end-plate sensitivity to acetylcholine in muscles. It could induce serious arrhythmia, myocardial depression, and vasodilation, which cause hypotension (34). Finally, according to the results of subgroup analyses, we should realize that high admission serum magnesium levels should be paid more attention in patients with younger age (<67 years), combined with cardiac arrest and higher Hb (>13.1 g/dl); higher RBC (>3.96 m/μl) and higher total calcium (≥8.6 mg/dl) subgroup; and lower BUN (<21 mg/dl) and potassium (<4.3 mmol/L). The synergistic effect with potassium and antagonistic effects with calcium is widely discussed in previous studies (35, 36). Hence, in patients with other electrolyte imbalances, high-normal serum magnesium should be paid more attention. Moreover, serum magnesium levels are closely associated with renal function and renal excretion, but a higher risk of mortality in the lower BUN subgroup was rarely reported before. Finally, as the previous study reported, a magnesium deficiency could cause increased intestinal absorption of iron and decreased erythrocyte counts. Hence, the higher risk in higher RBC and Hb subgroup caused by increase in Mg probably accounts for the increase in erythrocyte and Hb concentrations.

Interestingly, high-normal serum magnesium and hypermagnesemia are not associated with 30-day in-hospital mortality in the subgroup of those with cardiogenic shock and epinephrine. Generally, patients with cardiogenic shock and epinephrine always have combined severe cardiovascular complications and various electrolyte and arterial blood gas disturbance. Due to the insignificant contribution to the risk of mortality, the correction of serum could be of secondary importance in these patients. Moreover, we also found that Mg supplementation did not affect the prediction of high-normal serum magnesium and hypermagnesemia for in-hospital mortality. Hence, the benefits of short-term in-hospital magnesium supplementation should be rethought in further clinical studies in critically ill patients with AMI.

Our study has convenient clinical implications. Serum magnesium in blood electrolyte routine could serve as a simple and quick biomarker for predicting in-hospital mortality of AMI. Moreover, the main strength of this study is the huge population size of more than 9,000 patients from a multicenter EHR. Moreover, these data were collected systematically. To our knowledge, the scale of the population size makes this study of admission serum magnesium level in ICU patients with AMI one of the most comprehensive thus far. Although our findings cannot provide a more appropriate recommended serum magnesium level, caution should be taken to not overcorrect magnesium levels especially in AMI patients in the ICU.

However, this study still has several limitations. Firstly, pre-hospital information such as medication is lacking, which could affect the level of serum magnesium. Additionally, although we had made every effort to adjust to potential confounding factors through multivariate logistic analysis, there still remained other in-hospital variables that could confuse the predicted effect of serum magnesium. Finally, due to the limited data from the dataset, we could not obtain the serum magnesium concentration at the timing of life-threatening events, so the direct association between the serum magnesium and life-threatening events could not be illustrated in this study (37). Therefore, larger, more complete studies are needed to be conducted to confirm our results.

## Conclusion

High-normal serum magnesium (>2.2, ≤2.4 mg/dl) and hypermagnesemia (>2.4 mg/dl) are both independent predictors of 30-day in-hospital mortality in ICU patients with AMI, indicating that serum magnesium might have the potential to be a useful prognostic biomarker for AMI.

## Data Availability Statement

Publicly available datasets were analyzed in this study. This data can be found at the eICU-Collaborative Research Database.

## Author Contributions

RS: conceptualization, methodology, and supervision. LT: methodology, software, data curation, visualization, investigation, and original draft preparation. QX: original draft preparation and revision and reviewing and editing. CL: original draft preparation and revision reviewing and editing. JL: data curation and original draft preparation. All authors listed have made a substantial, direct and intellectual contribution to the work, and approved it for publication.

## Conflict of Interest

The authors declare that the research was conducted in the absence of any commercial or financial relationships that could be construed as a potential conflict of interest.
