# Supplementary material for: High-Normal Serum Magnesium and Hypermagnesemia Are Associated With Increased 30-Day In-Hospital Mortality: A Retrospective Cohort Study
Source: Front Cardiovasc Med. 2021 Feb 10;8:625133. doi: 10.3389/fcvm.2021.625133 (PMC7902876; doi:10.3389/fcvm.2021.625133)
Supplement: Supplementary file 1 [file Table_1.DOCX]

**Supplymental Table1:** Subgroup analysis of the associations between serum magnesium levels and in-hospital mortality in cox regression model.

| Variables | Serum Mg levels (mmol/L) | | | | | Interaction  P value |
| --- | --- | --- | --- | --- | --- | --- |
|  | <1.8 | ≥1.8; <2.0 | ≥2.0 ,  ≤ 2.2 | >2.2; ≤2.4 | >2.4 |  |
| Gender  Female  Male  Age,years  <67  ≥67  Ethnicity  African American  Asian  Caucasian  Hispanic  Native American  Others  Hypertension  No  Yes  CHF  No  Yes  Diabetes  No  Yes  PVD  No  Yes  Prior MI  No  Yes  Atrial fibrillation  No  Yes  Valvular disease  No  Yes  Cardiogenic shock  No  Yes  Cardiac arrest  No  Yes  HC  No  Yes  Norepinephrine  No  Yes  Dopamine  No  Yes  Epinephrine  No  Yes  Mg supplyment  No  Yes  HR (beats/min)  <83  ≥83  SBP, mmHg  <122  ≥122  DBP, mmHg  <70  ≥70  MBP, mmHg  <88  ≥88  WBC, K/uL  <12.3  ≥12.3  Platelet, K/uL  <223  ≥223  Hb, (g/dL)  <13.1  ≥13.1  RBC, K/uL  <3.96  ≥3.96  BUN, mg/dL  <21  ≥21  Cr, mg/dL  <1.17  ≥1.17  Glucose, mg/dL  <158  ≥158  Potassium, mmol/L  <4.3  ≥4.3  Sodium, mmol/L  <140  ≥140  Bicarbonate,mmol/L  <25  ≥25  Calcium,mg/dL  <8.6  ≥8.6 | 1.31 (1.00, 1.72)  0.89 (0.69, 1.16)  1.16 (0.82, 1.66)  1.07 (0.86, 1.33)  1.73 (0.82, 3.62)  1.20 (0.41, 3.46)  1.06 (0.86, 1.30)  2.29 (1.04, 5.06)  /  0.63 (0.18, 2.18)  0.90 (0.63, 1.29)  1.18 (0.95, 1.47)  1.06 (0.84, 1.34)  1.13 (0.84, 1.53)  1.22 (0.99, 1.52)  0.81 (0.56, 1.16)  1.09 (0.90, 1.33)  1.08 (0.59, 2.00)  1.10 (0.90, 1.35)  1.06 (0.68, 1.63)  1.17 (0.94, 1.45)  0.88 (0.61, 1.27)  1.08 (0.90, 1.31)  1.46 (0.65, 3.29)  1.09 (0.89, 1.34)  1.15 (0.77, 1.74)  1.01 (0.81, 1.25)  1.64 (1.13, 2.39)  1.14 (0.94, 1.38)  0.60 (0.25, 1.42)  1.10 (0.89, 1.37)  1.16 (0.80, 1.69)  1.10 (0.91, 1.32)  1.40 (0.24, 8.25)  1.09 (0.90, 1.31)  1.41 (0.64, 3.10)  1.06 (0.85, 1.39)  1.17 (0.84, 1.49)  0.93 (0.66, 1.30)  1.12 (0.90, 1.40)  1.10 (0.87, 1.40)  1.07 (0.80, 1.44)  1.07 (0.84, 1.36)  1.12 (0.84, 1.49)  1.13 (0.89, 1.42)  1.03 (0.76, 1.40)  1.30 (0.92, 1.85)  1.01 (0.81, 1.25)  1.12 (0.86, 1.46)  1.07 (0.83, 1.39)  1.02 (0.80, 1.29)  1.21 (0.90, 1.63)  0.98 (0.78, 1.23)  1.36 (0.99, 1.88)  1.69 (1.09, 2.62)  1.00 (0.81, 1.23)  1.24 (0.85, 1.81)  1.03 (0.83, 1.27)  1.16 (0.83, 1.61)  1.02 (0.82, 1.28)  1.26 (0.91, 1.73)  1.04 (0.83, 1.30)  1.04 (0.79, 1.36)  1.15 (0.89, 1.49)  0.95 (0.75, 1.20)  1.13 (0.83, 1.52)  0.98 (0.78, 1.23)  1.25 (0.91, 1.72) | 0.94 (0.70, 1.26)  0.77 (0.60, 0.99)  0.95 (0.67, 1.37)  0.79 (0.63, 0.99)  0.75 (0.28, 1.99)  0.18 (0.02, 1.38)  0.84 (0.68, 1.03)  1.52 (0.70, 3.28)  /  0.83 (0.35, 1.99)  0.89 (0.65, 1.22)  0.81 (0.64, 1.03)  0.85 (0.67, 1.08)  0.80 (0.58, 1.10)  0.93 (0.75, 1.16)  0.60 (0.40, 0.89)  0.86 (0.71, 1.05)  0.58 (0.29, 1.17)  0.86 (0.69, 1.06)  0.75 (0.48, 1.18)  0.94 (0.76, 1.17)  0.57 (0.38, 0.85)  0.84 (0.69, 1.03)  0.75 (0.34, 1.64)  0.84 (0.68, 1.04)  0.90 (0.59, 1.38)  0.81 (0.65, 1.00)  1.04 (0.70, 1.55)  0.82 (0.67, 1.00)  1.13 (0.52, 2.43)  0.83 (0.66, 1.03)  0.94 (0.65, 1.36)  0.85 (0.70, 1.02)  /  0.86 (0.71, 1.04)  0.57 (0.21, 1.55)  0.82 (0.77, 1.00)  1.23 (0.62, 2.43)  0.79 (0.58, 1.09)  0.86 (0.68, 1.09)  0.93 (0.74, 1.18)  0.67 (0.49, 0.93)  0.88 (0.69, 1.12)  0.75 (0.55, 1.03)  0.89 (0.70, 1.12)  0.74 (0.53, 1.02)  1.13 (0.80, 1.60)  0.74 (0.59, 0.93)  0.78 (0.59, 1.03)  0.90 (0.69, 1.17)  0.87 (0.68, 1.10)  0.77 (0.56, 1.06)  0.88 (0.70, 1.10)  0.72 (0.50, 1.04)  1.22 (0.78, 1.92)  0.80 (0.65, 0.98)  0.85 (0.56, 1.28)  0.83 (0.67, 1.03)  0.77 (0.54, 1.09)  0.87 (0.69, 1.09)  0.80 (0.56, 1.13)  0.87 (0.69, 1.09)  0.74 (0.55, 0.98)  0.93 (0.72, 1.20)  0.78 (0.61, 1.01)  0.87 (0.64, 1.17)  0.82 (0.65, 1.04)  0.85 (0.62, 1.19) | ref  ref  ref  ref  ref  ref  ref  ref  ref  ref  ref  ref  ref  ref  ref  ref  ref  ref  ref  ref  ref  ref  ref  ref  ref  ref  ref  ref  ref  ref  ref  ref  ref  ref  ref  ref  ref  ref  ref  ref  ref  ref  ref  ref  ref  ref  ref  ref  ref  ref  ref  ref  ref  ref  ref  ref  ref  ref  ref  ref  ref  ref  ref  ref  ref  ref  ref  ref | 1.97 (1.46, 2.67)  1.40 (1.08, 1.80)  1.61 (1.10, 2.35)  1.60 (1.27, 2.00)  2.85 (1.32, 6.16)  0.47 (0.06, 3.63)  1.58 (1.27, 1.96)  2.74 (1.12, 6.67)  1.55 (0.09, 26.48)  1.25 (0.45, 3.47)  1.36 (0.95, 1.97)  1.71 (1.36, 2.16)  1.54 (1.20, 1.98)  1.61 (1.19, 2.19)  1.71 (1.35, 2.15)  1.36 (0.95, 1.94)  1.60 (1.31, 1.96)  1.57 (0.79, 3.11)  1.61 (1.29, 1.99)  1.57 (1.01, 2.44)  1.67 (1.32, 2.10)  1.34 (0.93, 1.93)  1.55 (1.26, 1.90)  2.18 (1.11, 4.29)  1.65 (1.33, 2.05)  1.41 (0.90, 2.22)  1.55 (1.24, 1.93)  1.69 (1.13, 2.52)  1.53 (1.25, 1.88)  2.80 (1.40, 5.60)  1.54 (1.22, 1.94)  1.60 (1.13, 2.28)  1.61 (1.32, 1.96)  0.81 (0.14, 4.74)  1.63 (1.34, 1.99)  0.88 (0.37, 2.10)  1.54 (1.15, 1.89)  2.81 (1.52, 4.60)  1.68 (1.23, 2.32)  1.53 (1.19, 1.95)  1.69 (1.31, 2.17)  1.50 (1.11, 2.04)  1.61 (1.26, 2.07)  1.55 (1.14, 2.11)  1.63 (1.27, 2.08)  1.54 (1.12, 2.12)  2.03 (1.40, 2.94)  1.39 (1.11, 1.75)  1.58 (1.19, 2.08)  1.63 (1.24, 2.13)  1.53 (1.19, 1.95)  1.70 (1.24, 2.33)  1.45 (1.15, 1.83)  1.97 (1.39, 2.79)  1.74 (1.02, 2.97)  1.48 (1.21, 1.83)  1.35 (0.84, 2.16)  1.51 (1.22, 1.87)  1.96 (1.39, 2.78)  1.37 (1.08, 1.73)  1.57 (1.07, 2.28)  1.56 (1.24, 1.96)  1.58 (1.18, 2.10)  1.62 (1.24, 2.11)  1.34 (1.03, 1.73)  1.89 (1.41, 2.53)  1.47 (1.16, 1.87)  1.81 (1.31, 2.52) | 1.80 (1.32,2.45)  1.51 (1.18,1.93)  2.24 (1.59,3.17)  1.37 (1.09,1.73)  2.78 (1.25,6.20)  0.45 (0.10,2.05)  1.65 (1.34,2.04)  0.94 (0.36,2.47)  /  2.13 (0.86,5.30)  1.61 (1.15,2.24)  1.61 (1.28,2.04)  1.76 (1.39,2.23)  1.35 (0.97,1.88)  1.77 (1.42,2.22)  1.23 (0.84,1.79)  1.64 (1.34,2.00)  1.28 (0.63,2.60)  1.68 (1.36,2.07)  1.30 (0.81,2.09)  1.74 (1.40,2.18)  1.25 (0.85,1.82)  1.60 (1.31,1.95)  1.66 (0.80,3.42)  1.77 (1.43,2.19)  0.92 (0.58,1.45)  1.36 (1.08,1.71)  1.96 (1.37,2.81)  1.62 (1.33,1.97)  1.49 (0.69,3.21)  1.56 (1.24,1.96)  1.62 (1.13,2.31)  1.66 (1.37,2.01)  /  1.69 (1.39,2.06)  0.39 (0.13,1.21)  1.36 (1.08,1.71)  1.76 (1.37,2.81)  1.72 (1.26,2.36)  1.52 (1.19,1.94)  1.58 (1.24,2.01)  1.58 (1.15,2.16)  1.68 (1.32,2.14)  1.46 (1.07,1.99)  1.64 (1.29,2.08)  1.46 (1.05,2.03)  1.75 (1.19,2.58)  1.47 (1.18,1.83)  1.76 (1.35,2.28)  1.44 (1.08,1.92)  1.35 (1.05,1.74)  2.07 (1.54,2.77)  1.20 (0.94,1.52)  3.02 (2.19,4.17)  3.07 (1.94,4.86)  1.30 (1.05,1.61)  1.65 (1.06,2.55)  1.44 (1.16,1.78)  1.45 (1.00,2.12)  1.56 (1.25,1.95)  2.09 (1.47,2.96)  1.40 (1.11,1.76)  1.46 (1.08,1.96)  1.71 (1.33,2.20)  1.51 (1.18,1.93)  1.56 (1.15,2.12)  1.32 (1.04,1.68)  2.15 (1.55,2.98) | 0.270  0.168  0.272  0.495  0.544  0.206  0.766  0.895  0.230  0.781  0.018  0.201  0.103  0.985  0.045  0.017  0.089  0.549  0.497  0.876  0.907  0.276  0.690  0.147  <0.001  0.009  0.737  0.248  0.179  0.756  0.440  0.160 |
